# Bromodomain and Extra-Terminal Family Proteins BRD2, BRD3, and BRD4 Contribute to H19-Dependent Transcriptional Regulation of Cell Adhesion Molecules, Modulating Metastatic Dissemination Program in Prostate Cancer

**DOI:** 10.3390/ncrna11030033

**Published:** 2025-04-29

**Authors:** Valeria Pecci, Melissa Borsa, Aurora Aiello, Sara De Martino, Luca Cis, Cristian Ripoli, Dante Rotili, Francesco Pierconti, Francesco Pinto, Claudio Grassi, Carlo Gaetano, Antonella Farsetti, Simona Nanni

**Affiliations:** 1 Department of Translational Medicine and Surgery, Università Cattolica del Sacro Cuore, 00168 Rome, Italy; valeria.pecci@unicatt.it (V.P.); melissa.borsa@unicatt.it (M.B.); luca.cis@unicatt.it (L.C.); 2 National Research Council (CNR)-IASI, 00185 Rome, Italy; aurora.aiello@cnr.it (A.A.); sar.demartino@gmail.com (S.D.M.); 3 Fondazione “Policlinico Universitario A. Gemelli IRCCS”, 00168 Rome, Italy; cristian.ripoli@unicatt.it (C.R.); francesco.pierconti@unicatt.it (F.P.); francesco.pinto@unicatt.it (F.P.); claudio.grassi@unicatt.it (C.G.); 4 Department of Neuroscience, Università Cattolica del Sacro Cuore, 00168 Rome, Italy; 5 Dipartimento di Chimica e Tecnologie del Farmaco, Sapienza Università di Roma, 00185 Rome, Italy; dante.rotili@uniroma3.it; 6 Department of Woman, Child and Public Health, Università Cattolica del Sacro Cuore, 00168 Rome, Italy; 7 Laboratory of Epigenetics, Istituti Clinici Scientifici Maugeri IRCCS, 27100 Pavia, Italy; carlo.gaetano@icsmaugeri.it

**Keywords:** long non-coding RNA, metastatic program, epigenetics, transcription regulation

## Abstract

Background/Objectives: Metastatic prostate cancer (PCa) remains a major clinical challenge with limited therapeutic options. The long non-coding RNA *H19* has been implicated in regulating cell adhesion molecules and collective migration, key features of metastatic dissemination. This study investigates the role of the Bromodomain and Extra-Terminal (BET) proteins BRD2, BRD3, and BRD4 in the *H19*-dependent transcriptional regulation of cell adhesion molecules. Currently, the major effects of BET inhibitors require androgen receptor (AR) expression. Methods: *H19* was stably silenced in PC-3 (AR-null) and 22Rv1 (AR-positive) castration-resistant PCa cells. The cells were treated with the pan-BET inhibitors JQ1 and OTX015 or the BET degrader dBET6. In vivo, the effects of JQ1 were evaluated in xenograft mouse models. Chromatin immunoprecipitation (ChIP) and RNA-ChIP were used to assess BET protein recruitment and interaction with cell adhesion gene loci and *H19*. Organotypic slice cultures (OSCs) from fresh PCa surgical specimens were used as ex vivo models to validate transcriptional changes and BRD4 recruitment. Results: BET inhibition significantly reduced the expression of β4 integrin and E-cadherin and cell proliferation in both basal conditions, and following *H19* knockdown in PC-3 and 22Rv1 cells. These effects were mirrored in JQ1-treated tumor xenografts, which showed marker downregulation and tumor regression. ChIP assays revealed that BRD4, more than BRD2/3, was enriched on β4 integrin and E-cadherin promoters, especially in regions marked by H3K27ac. *H19* silencing markedly enhanced BRD4 promoter occupancy. RNA-ChIP confirmed a specific interaction between BRD4 and *H19*. These findings were validated in OSCs, reinforcing their clinical relevance. Conclusions: Our study demonstrates that BRD4 epigenetically regulates the *H19*-mediated transcriptional control of adhesion molecules involved in collective migration and metastatic dissemination. Importantly, these effects are independent of AR status, suggesting that targeting the *H19*/BRD4 axis may represent a promising therapeutic avenue for advanced PCa.

## 1. Introduction

Prostate cancer (PCa) is one of the most common malignancies and a leading cause of cancer-related death among men worldwide [1,2]. While early-stage PCa is often successfully treated with surgery, radiation, and androgen deprivation therapy, about 30% of cases progress to metastatic disease, for which curative treatment remains elusive [3,4]. Understanding the molecular mechanisms driving metastatic dissemination beyond androgen receptor (AR) signaling is essential for developing alternative therapeutic strategies. Metastatic dissemination in PCa involves dynamic tumor cell migration and invasion strategies, primarily through single-cell or collective cell migration modes detaching from tumor mass to actively move and invade surrounding tissues. Single-cell dissemination often correlates with the epithelial-to-mesenchymal transition (EMT), a process marked by the downregulation of E-cadherin, increased motility, and invasiveness. In contrast, collective migration, or cohesive metastatic phenotype, involves clusters of cancer cells that retain cell–cell junctions and simultaneously adhere to the extracellular matrix (ECM) via β integrin signaling. This model is characterized by the sustained expression of E-cadherin and β integrin subunits [5,6,7,8].

Recent studies emphasize the role of epigenetic regulators in orchestrating these migratory programs. Among them, the bromodomain and extra-terminal domain (BET) family proteins—BRD2, BRD3, and BRD4—act as key epigenetic readers that bind acetylated lysine residues on histones and non-histone proteins [9,10]. By facilitating the chromatin recruitment of transcriptional regulators such as P-TEFb, BETs contribute to transcription initiation and the elongation of various oncogenic targets, including *c-MYC*, *CCND1*, and *JUNB* [11,12,13]. BRD4 represents the most extensively characterized member, acting as a global transcriptional coactivator. Comprehensively, targeting BET proteins is appealing for designing efficacious cancer treatment drugs. Accordingly, in recent years, a variety of small-molecule BET inhibitors (BETis) have been developed, including pan-BETis such as JQ1 (thieno-triazolo-1,4-diazepine) and its analogs (OTX015), firstly reported by Filippakopoulos et al. [14]; proteolysis-targeting chimeras (PROTACs); chimeric bifunctional small molecules that recruit an E3 ligase to promote the degradation of a target protein; and the more recently discovered BRD4-selective inhibitors [15]. Unfortunately, the clinical effectiveness of the present BETi is limited, prompting the exploration of novel approaches like the development of Dual-Target BET inhibitors [10,16].

The BET proteins (BRD2/3/4) are key epigenetic co-regulators mainly for prostate cancer growth, with BRD4 being a critical component of AR signaling [17,18]. Indeed, it has been shown that JQ1 abrogated BRD4 localization to AR target loci and AR-mediated gene transcription [17]. Similarly, BRD4 is recruited in breast cancer cells on promoters of estrogen receptor alpha (ERα)-dependent genes following estrogen stimulation to regulate estrogen-induced transcription [19].

Regarding advanced forms of PCa, even castration-resistant ones, the significant effects of BETis have been attributed to AR activity and signaling, with initial preclinical studies finding them less effective in AR-null versus AR-positive prostate cancer [17,20,21,22]. Notably, although BETis were initially considered applicable, primarily in AR-positive PCa, emerging evidence supports a broader role, including in AR-null neuroendocrine subtypes [23]. This observation suggests the involvement of AR-independent transcriptional programs regulated by BETs, which remain to be fully elucidated.

Long non-coding RNAs (lncRNAs) have emerged as important epigenetic and transcriptional regulators in cancer biology. One of the first discovered, *H19*, encodes a 2.6 kb polyadenylated lncRNA expressed during embryonic development and aberrantly reactivated in multiple cancers [24,25,26]. Mechanistically, *H19* mediates diverse regulatory functions by acting as a competing endogenous RNA (ceRNA) and a sponge for microRNAs, and as a modular scaffold for several RNA binding proteins [e.g., KH-Type Splicing Regulatory Protein (KHSRP) and Heterogeneous Nuclear Ribonucleoprotein U (HnRNP U)] and DNA/chromatin modification factors [Polycomb Repressive Complex 2 (PRC2) and Methyl-CpG–Binding Domain Protein 1 (MBD1)], highlighting a crucial role of *H19* on gene expression modulation [24,25]. Functionally, *H19* presents a dual nature, acting as both an oncogene and a tumor suppressor gene, depending on cancer type and tumor microenvironment. This context-dependent behavior of *H19* emphasizes its multiple roles in cancer biology with a complex array of mechanisms [26,27].

*H19*’s relevance in PCa is supported by studies demonstrating that its downregulation contributes to a switch toward the collective metastatic phenotype via the upregulation of E-cadherin and β4 integrin [28,29,30]. Furthermore, *H19* is transcriptionally repressed by AR signaling, as reported in neuroendocrine PCa models [31]. These findings place *H19* at the intersection of hormone signaling, epigenetic regulation, and metastasis.

The tumor microenvironment also plays a pivotal role in modulating PCa progression and EMT-related plasticity. Epigenetic reprogramming within the microenvironment alters chromatin organization and transcriptional outputs, influencing dissemination strategies [32,33]. Building on our previous work identifying the *H19*/cell adhesion molecule circuitry as a driver of collective migration [29,30,34], we now explore how BET family proteins contribute to this pathway. Specifically, we evaluate the molecular interactions between BRD2, BRD3, BRD4, and *H19* and their role in the transcriptional regulation of adhesion genes in both AR-null and AR-positive PCa contexts.

## 2. Results

### 2.1. BET Family Proteins BRD2, BRD3 and BRD4 Are Involved in Regulation of H19/Cell Adhesion Molecules Circuitry

In prostate cancer, we molecularly characterized the *H19*/cell adhesion molecules’ circuitry, in which *H19* acts as a transcriptional repressor of specific cell adhesion molecules, including E-cadherin and β4 integrin, by recruiting the EZH2 polycomb subunit on the promoter region and increasing the H3K27me3 level. Specific pro-tumoral stimuli, like estrogen and hypoxia, drive a peculiar reduction in the H19 level, leading to an increase in both E-cadherin and β4 integrin, thus activating the collective cell migration program and metastatic dissemination [28,29,30,34].

**Figure 1:**
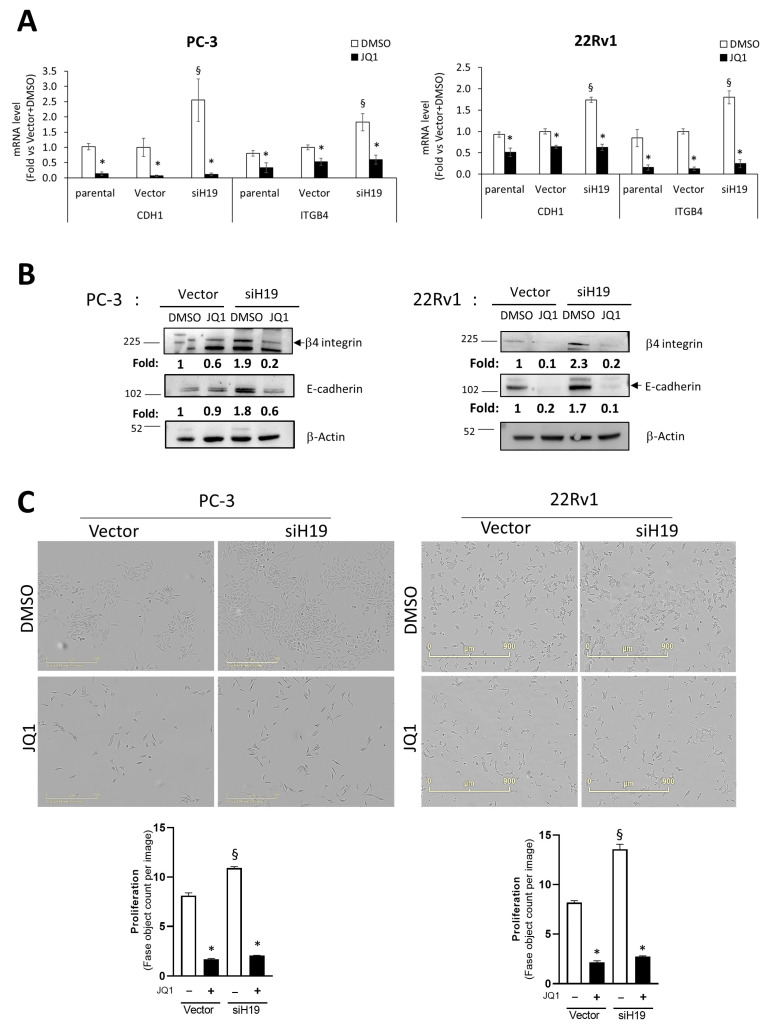
Modulation of E-cadherin and β4 integrin level and effect on cell proliferation mediated by BET bromodomain family protein inhibitor JQ1. (**A**) E-cadherin (*CDH1*), and β4 integrin (*ITGB4*) mRNAs were assessed by qPCR in PC-3-luc (PC-3, left) and 22Rv1-luc (22Rv1, right) after stable *H19* silencing (siH19) compared to control vector (Vector) or parental cells in presence or absence of 72 h treatment with JQ1 inhibitor or DMSO as control. Data, plotted as fold change vs. Vector + DMSO, represent mean ± SEM of 4 independent experiments. (**B**) Representative E-Cadherin and β4-integrin Western blot. β-Actin served as control. Molecular weight marker is indicated. Number represents densitometric analysis of protein level normalized to β-Actin and expressed as fold change vs. Vector + DMSO. (**C**) Cell proliferation was monitored using the IncuCyte live cell analysis system. Upper: raw data pictures of cell confluence exported from IncuCyte system after 48 h incubation; scale bar is indicated. Lower: Cell confluence was calculated from raw data images; data represent mean ± SEM of 3 independent experiments, each performed in triplicate. * *p* < 0.05 JQ1 vs. DMSO; § *p* < 0.05 siH19 vs. Vector.

To assess whether the BET family proteins BRD2, BRD3, and BRD4 are involved in the regulation of the above *H19*/cell adhesion molecules’ circuitry, the PC-3 and 22Rv1 prostate cancer cell lines were treated with JQ1, a potent inhibitor of the BET family of acetyl-lysine recognition motifs, including the BRD2, BRD3, BRD4, and BRDT bromodomains. The expression level of the cell adhesion molecules E-cadherin and β4 integrin was evaluated in stable *H19*-silenced cells (siH19, *H19* depletion range 75–85%, as previously described in [30]) compared to the control vector (Vector) or parental cells (Figure 1). As expected, E-cadherin and β4 integrin cell adhesion molecules were induced at the mRNA and protein levels after *H19* silencing compared to the vector (Figure 1A and Figure 1B, respectively). Notably, JQ1 was effective in reducing cell adhesion molecules not only in siH19 cells, but also in the control vector and parental cells, and in inhibiting cell proliferation (Figure 1C).

**Figure 2:**
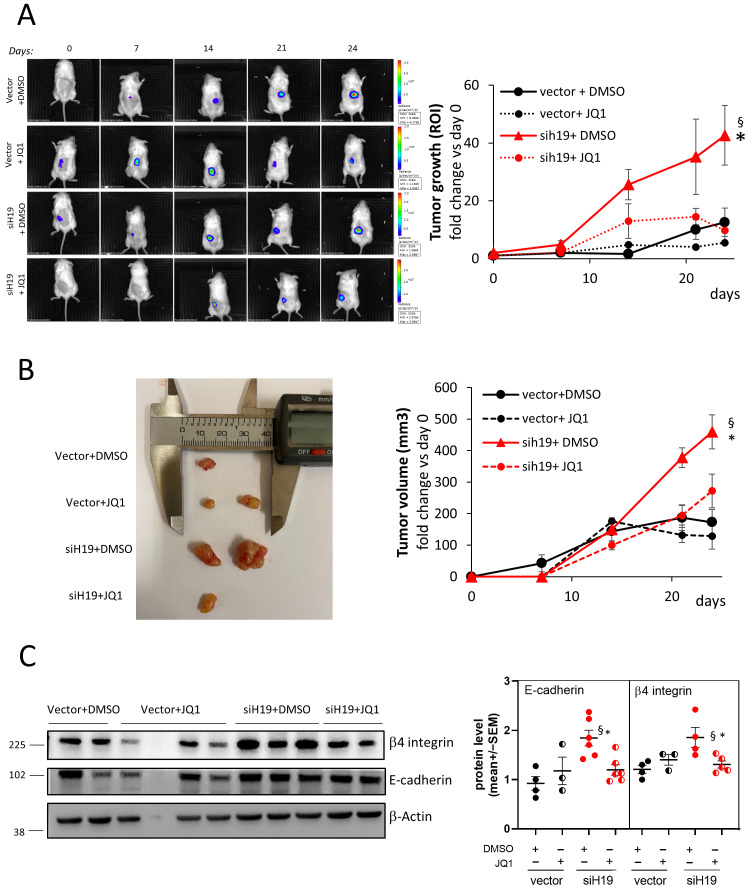
Effect of JQ1 treatment on a subcutaneous murine xenograft tumor. (**A**) Sequential in vivo imaging of tumor growth post subcutaneous injection of *H19*-silenced PC-3-luc (siH19) and control vector cells in NOD/SCID mice treated with JQ1 (25 mg/kg) or vehicle (DMSO). Panels depict a representative mouse from each group (**left**). Tumor growth was measured as photons/sec in the region of interest (ROI). Data, plotted as fold change vs. day 0, represent mean ± SEM of 6 mice/group (**right**). (**B**) Ex vivo photos of representative solid tumors on the day of the explant (**left**). Tumor volume was evaluated by caliper measurements at the different time points and calculated as follows: V = (w^2^ × l)/2; w = width, l = length. Data represent mean ± SEM of 6 mice/group (**right**). (**C**) β4 integrin and E-cadherin protein level analyzed by Western blot in tumor samples. β-Actin was used as a loading control. Molecular weight marker is indicated. * *p* < 0.05 vs. Vector + DMSO; § *p* < 0.05 vs. siH19 + JQ1.

Of note, the reduction in E-cadherin and β4 integrin expression following JQ1 treatment was also observed in vivo in tumor xenograft mice obtained with the subcutaneous injection of *H19*-silenced PC-3-luc (siH19) and control vector cells in NOD/SCID mice (Figure 2C). In parallel, a significant reduction in tumor growth and tumor size was observed upon JQ1 treatment (Figure 2A and Figure 2B, respectively).

**Figure 3:**
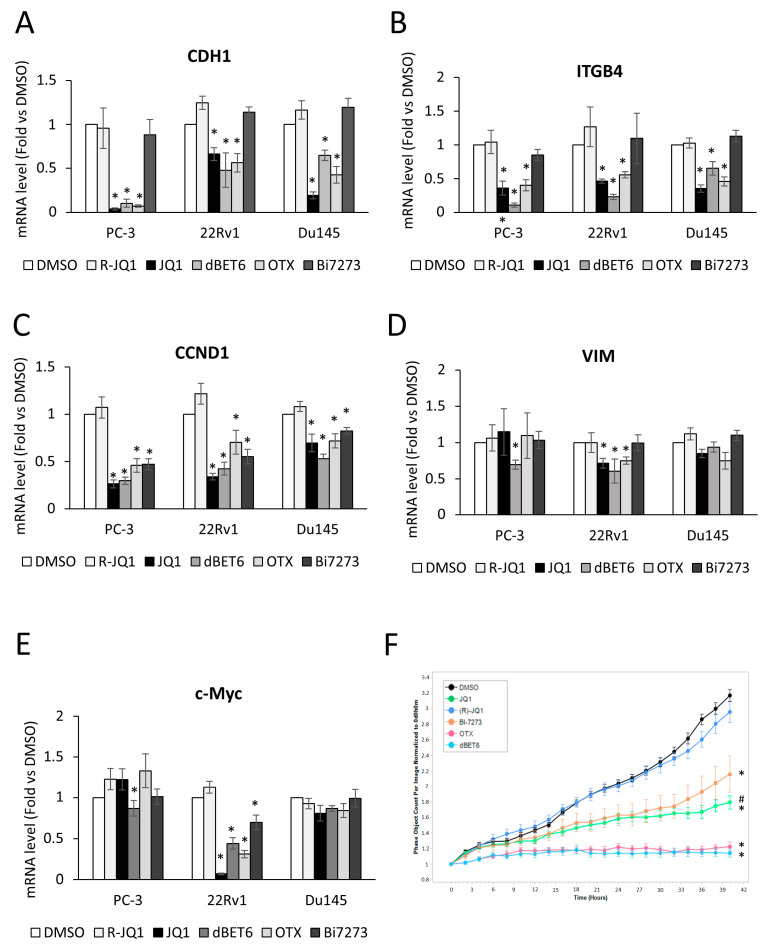
Effect of several BET family protein inhibitors, BRD2/3/4 or BRD7/9 members, on the gene expression level of E-cadherin and β4 integrin and cell proliferation. E-cadherin (*CDH1*, (**A**)), β4 integrin (*ITGB4*, (**B**)), Cyclin D1 (*CCND1*, (**C**)), Vimentin (*VIM*, (**D**)), and c-*Myc* (**E**) mRNAs were assessed by qPCR in PC-3-luc (PC-3), 22Rv1-luc (22Rv1), and Du145 in presence or absence of 48 h treatment with BRD2/3/4 family inhibitors, JQ1, dBET6, OTX015 (OTX), or BRD7/9, inhibitor Bi7273, or inactive (R)-(-)-JQ1 (R-JQ1), or DMSO as control. Data, plotted as fold change vs. DMSO, represent the mean ± SEM of 4 independent experiments. * *p* < 0.05 vs. DMSO. (**F**) PC-3 cell proliferation was monitored using the IncuCyte live cell analysis system. Cell confluence was calculated from raw data images; the data shown is a representative experiment of 4 biological replicates, and each time point represents mean ± SEM of 4 samples. * *p* < 0.05 vs. DMSO; # *p* < 0.05 vs. (R)-JQ1.

To gain a deeper insight into BRD’s involvement in E-cadherin and β4 integrin gene expression, PC-3 and 22Rv1 cells were also treated with the BET inhibitors OTX015 (birabresib) or the BET degrader dBET6, showing similar results compared to JQ1 in reduction in *CDH1* and *ITGB4* (Figure 3A,B). No modulation was observed when the cells were treated, as a negative control, with the enantiomer (R)-(-)-JQ1 (R-JQ1)—the distomer of JQ1—with no effect on decreasing expression of BRD4-target genes. In addition, to evaluate the specificity of BRD2/3/4 involvement, we treated PCa-cells with the Bi7273 inhibitor that acts explicitly on BRD9, the BRD-containing subunit of the BAF (BRG-/BRM-associated factor), and its close homolog BRD7, showing excellent selectivity versus other BET family members [35]. As shown in Figure 3A,B, treatment with Bi7273 is ineffective in modulating cell adhesion molecules E-cadherin and β4 integrin, while it can downregulate a known BRD7/BRD9 target gene like *CCND1*. Notably, the downregulation of *CCND1* was also observed with BRD2/3/4 inhibitors JQ1, dBET6, and OTX015 (Figure 3C), while Vimentin was less sensitive to treatments (Figure 3D). Similar results were obtained in human prostate cancer Du145 cells, another androgen receptor-negative cell line derived from a PCa metastasis (Figure 3). Interestingly, JQ1, dBET6, and OTX015 are the most effective molecules in reducing cell proliferation compared to R-JQ1, or DMSO as a control (Figure 3F and Figure S1). In addition, reductions in *CDH1* and *ITGB4* expression and proliferation rate were also observed after treatment with LT052 [36], a highly selective inhibitor of the first bromodomain BD1 of the BET protein BRD4 (Figure S2).

Of note, the PC-3, Du145, and 22Rv1 cell lines represent models of advanced, androgen-independent prostate cancer with no androgen receptor (AR-null, PC-3, and Du145) expression or an androgen-insensitive AR variant (ARv7) co-expressed with a full-length AR (22Rv1). In line with previous results, a known direct BRD4 target gene, the driver oncogene c-Myc, was significantly repressed upon JQ1 treatments exclusively in the 22Rv1 cells, endogenously expressing the AR as consequence of a direct interaction between BRD4 and AR. In contrast, AR-null cells were almost insensitive to JQ1 and other BETis [11,12,13] (Figure 3E).

These results demonstrated that BET family proteins are strongly involved in the regulation of the *H19*/cell adhesion molecules’ circuitry, regardless of the androgen pathway.

### 2.2. E-Cadherin and β4 Integrin Are Direct Target Genes of the BET Family Members

**Figure 4:**
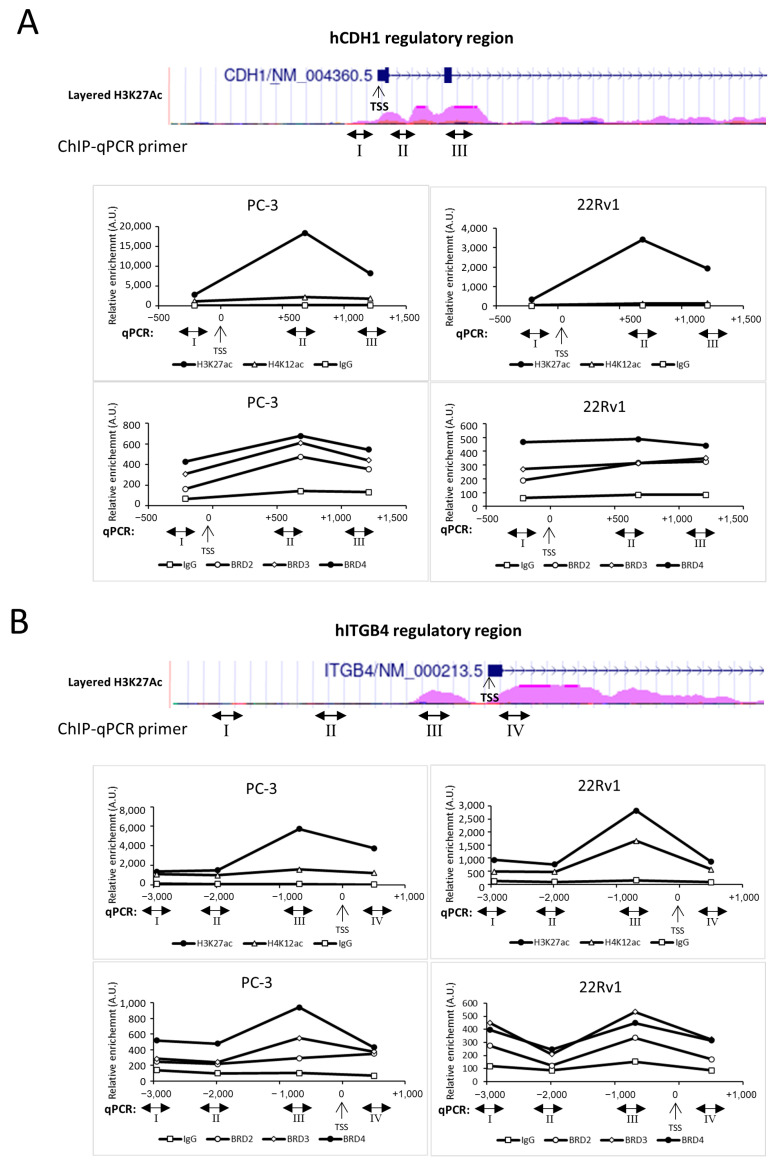
ChIPs onto promoter of *CDH1* and *ITGB4* cell-adhesion molecules. Upper panels: Schematic diagram of promoter and regulatory regions of *CDH1* (**A**) and *ITGB4* (**B**) genes. Violet peaks show enrichment of H3K27ac determined by the ChIP-seq assay from the ENCODE database (https://genome.ucsc.edu/cgi-bin/hgTrackUi?hgsid=2497230041_bmKILKoxbwq2oeOeS20t1RgmaAFn&c=chr16&g=wgEncodeRegMarkH3k27ac, accessed on 31 March 2025). Double-arrowed lines identify regions I, II, III, and IV, amplified by ChIP-qPCR. TSS: Transcription Start Site placed at 0 base pairs (bp). Lower panels: ChIP experiments were performed in parallel in PC-3-luc (PC-3) and 22Rv1-luc (22Rv1) cells. Immunoprecipitations were performed using antibodies to BRD2, BRD3, BRD4, H4K12ac, H3K27ac, or IgG as negative control. Data represent the means of 3 independent experiments normalized to input and plotted as relative enrichment in arbitrary units (A.U.). Roman numbers and TSS are as for panel A. Arabic numbers refer to the distance to TSS.

The BET bromodomain family proteins BRD2, BRD3, and BRD4 bind gene regulatory regions [37], frequently characterized by common markers like acetylation of H3K27 [38]. According to a ChIP-seq assay from the ENCODE database, the *CDH1* and *ITGB4* regulatory regions show H3K27 acetylation (H3K27ac) enrichment in both promoter and intron regions (Figure 4A,B). In line with this, JQ1 treatment decreases H3K27ac levels in our experimental models (Figure S3). Chromatin immunoprecipitation (ChIP) analyses were performed to evaluate whether E-cadherin and β4 integrin are direct target genes of the BET family members in both PC-3 and 22Rv1 cells. Chromatins were immunoprecipitated using antibodies to BRD2, BRD3, BRD4, H3K27ac, the acetylation of histone H4 at lysine 12 (H4K12ac), or IgG as a negative control, and DNA sequences proximal to or encompassing H3K27ac-enriched regions were amplified. H4K12ac modification was evaluated as alternative acetylated residues of histone H4 bound by BRD4 [39].

ChIP assays showed that BRD3 and BRD4 are primarily recruited in both *CDH1* and *ITGB4* regulatory regions compared to BRD2, especially in areas characterized by higher levels of H3K27ac: the first intron for *CDH1* (site II in Figure 4A) and the proximal promoter for *ITGB4* (site III in Figure 4B) regulatory regions. Notably, similar results were observed in the two cell lines analyzed, PC-3 and 22Rv1.

As a control, *c-Myc* and *IDO1* regulatory regions were analyzed in PC3 cells. As expected, mainly BRD4 was recruited on the *c-Myc* enhancer, with higher occupancy correlated with H3K27ac level (Figure S4A), while all BET family members, BRD2/3/4, similarly bound the IDO1 promoter paralleled by acetylated histone H3K27 (Figure S4B), as previously described in [40] and [41], respectively.

**Figure 5:**
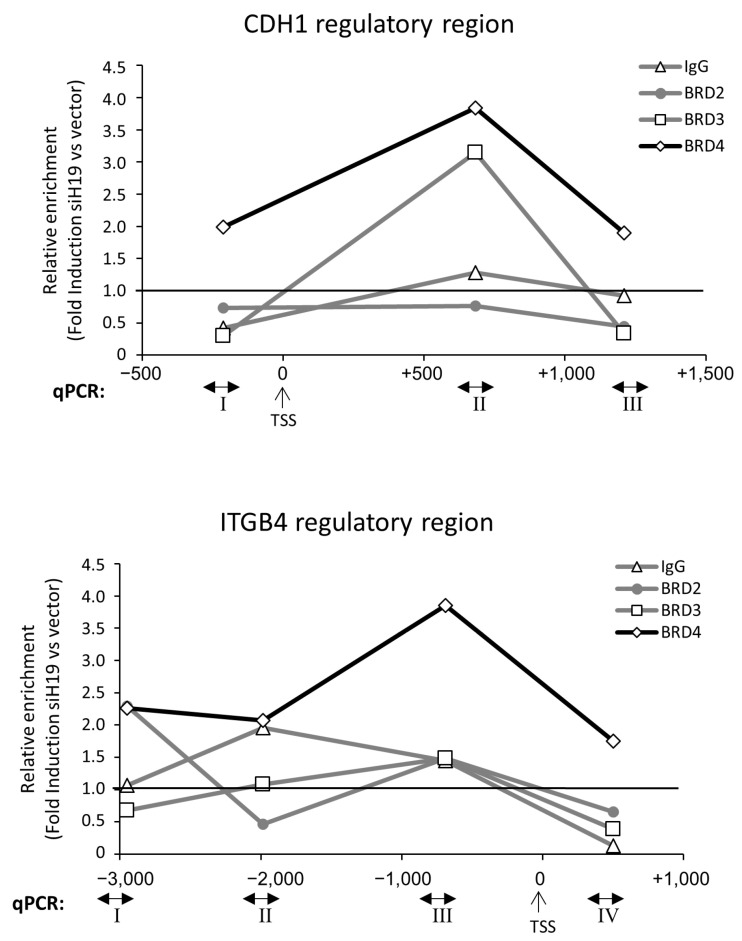
BRD2, BRD3, and BRD4 recruitment onto promoter of cell-adhesion molecules upon *H19* silencing. ChIP experiments were performed on PC-3 siH19 cells compared to vector control cells. Immunoprecipitations were performed using antibodies to BRD2, BRD3, BRD4, or IgG as negative controls. Data represent the means of 3 independent experiments normalized to input. Results are depicted as fold induction siH19 vs. Vector, taken as 1 (black line). Arabic numbers and primers for qPCR on *CDH1* regulatory regions (upper panel) or *ITGB4* regulatory region (lower panel) were as described in Figure 4. TSS = Transcriptional Strat Site.

To deepen insight into the molecular regulation of *H19*/cell adhesion molecules, ChIP assays were performed in siH19 cells compared to control vector cells (Figure 5). Interestingly, *H19* silencing perturbed BRD3 and BRD4 recruitment, specifically inducing occupancy in promoters. BRD4 recruitment was induced by *H19* silencing on both *CDH1* and *ITGB4* regulatory regions at all sites analyzed, ranging from 2- to 4-fold induction, while BRD3 recruitment was induced only at 1 intron in the *CDH1* gene (site II). On the other hand, BRD2 recruitment was not, or weakly, perturbed by siH19 in the *CDH1* and *ITGB4* regulatory regions, respectively.

**Figure 6:**
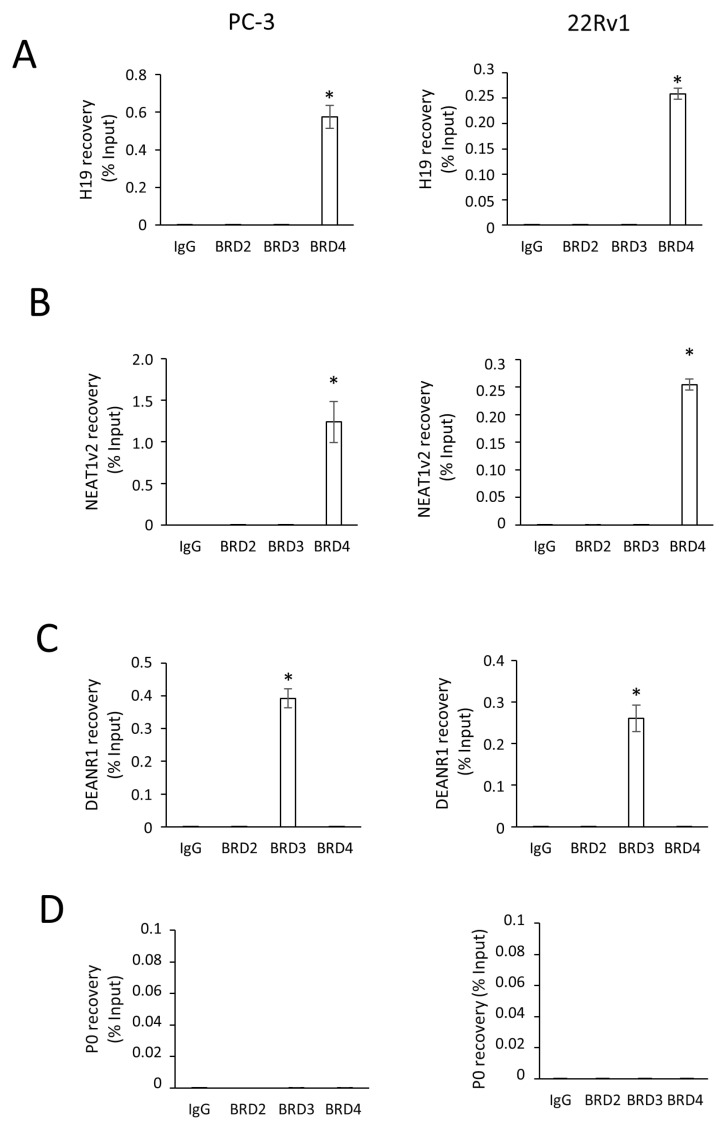
Analysis of *H19* interaction with BRD4 by RNA-ChIP. (**A**–**D**). *H19*, (**A**), *NEAT1v2* (**B**), *DEANR1* (**C**), and *P0* (**D**) interaction with BRD2, BRD3, or BRD4 detected by RNA-ChIP assays. RNA-ChIPs were performed using antibodies specific to BRD2, BRD3, or BRD4 in PC-3-luc (PC-3, left) and 22Rv1-luc (22Rv1, right) cells. IgG served as a negative control. Immunoprecipitated RNA was recovered and analyzed by *q*RT-PCR. Results are mean +/− SEM of 3 independent experiments. * *p* < 0.05 vs. IgG.

These results prompt us to hypothesize a direct interaction between BRDs and *H19* in both PC-3 and 22Rv1 cells. To test this hypothesis, the potential association between *H19* and BRDs on chromatin was investigated by RNA–Chromatin Immunoprecipitation (RNA-ChIP, Figure 6). RNA-ChIP experiments revealed a strong interaction between *H19* and BRD4, but not with BRD3 or BRD2 (Figure 6A). As a positive control, a BRD4 association with *NEAT1v2* (Figure 6B) was found [42], as well as a BRD3 association with *DEANR1* (Figure 6C, [43]). As a negative control, the housekeeping *P0* gene (Figure 6D) was used, and no interaction with BRD2, BRD3, or BRD4 was observed [42].

### 2.3. BRD3 and BRD4 Bind CDH1 and ITGB4 Regulatory Regions in Organotypic Slice Cultures (OSCs)

**Figure 7:**
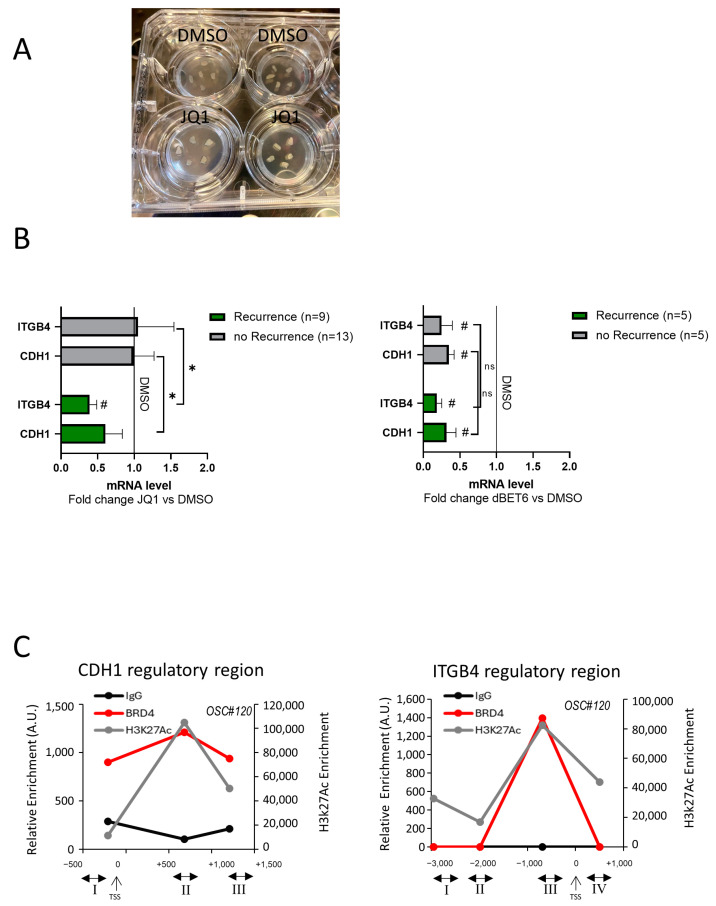
Modulation of E-cadherin and β4 integrin by JQ1 or dBET6 and in vivo ChIP assays on organotypic slices cultures. (**A**) Representative images of OSC after 72 h treatment with JQ1 or DMSO as control. (**B**) Quantification of *CDH1* and *ITGB4* transcripts in OSCs derived from PCa-patients with recurrence compared to no-recurrence group performed by qRT-PCR after 72 h treatment with JQ1 (**left**) or dBET6 (**right**) vs. DMSO as control. Data, plotted as fold change vs. DMSO (black line), represent mean ± SEM. # *p* < 0.05 vs. DMSO. Statistical significance between recurrence vs. no recurrence group was determined by the Chi-square test, 2-tail, on the proportion of the OSC with at least a 25% reduction in *CDH1* or *ITGB4* after JQ1 or dBET6 treatment. * *p* < 0.05. ns: not significant. (**C**) In vivo ChIP assays using fresh OSCs (n = 3; see also Figure S5). Immunoprecipitations were achieved with antibodies to BRD4 and H3K27ac or IgG as negative controls. Recruitment onto the *CDH1* and *ITGB4* regulatory regions was detected by quantitative PCR (qPCR) using primers for regions I, II, III, and IV, as in the legend to Figure 4.

To assess whether BRDs regulate *H19*/cell adhesion molecules in human prostate cancer tissues, human PCa-derived organotypic slice cultures (OSCs) were selected as a preclinical ex vivo model. OSCs, obtained from fresh explants of organ-confined prostate tumors during surgery, represent a relevant three-dimensional model recapitulating specific characteristics of the original tissue, also known as patient-derived tissue specimens [44,45] (Figure 7A). OSCs were obtained from a cohort of 22 PCa patients (Table 1) with localized disease undergoing surgery from November 2020 to June 2024 at the Urology of Università Cattolica (Rome, Italy). The disease’s clinical progression was defined by biochemical, local, or metastatic recurrence (n = 9 out of 22), with a follow-up of 7 months–4 years.

E-cadherin (*CDH1*) and β4 integrin (*ITGB4*) transcripts were assessed in OSCs treated for 72 h with JQ1 or dBET6 vs. vehicle (DMSO) by *q*RT-PCR (Figure 7B). After JQ1 addition, we observed a significant reduction in *ITGB4* but not *CDH1*, as compared to DMSO in OSCs derived from patients with recurrence. Interestingly, both *CDH1* and *ITGB4* mRNA reduction upon JQ1 was observed in OSCs derived from patients with recurrence compared to the no-recurrence group (Figure 7B, left). Of note, a significant reduction in both *CDH1* and *ITGB4* mRNAs was also observed in dBET6-treated OSCs as compared to DMSO. No difference has been noted between the recurrence (n = 5) compared to the no-recurrence (n = 5) group (Figure 7B, right).

Next, we asked whether the dynamic occupancy of the *CDH1* and *ITGB4* promoters by BRD4 and histone H3K27 acetylation also occurs in vivo. ChIP assays on OSC (n = 3) tissues were performed as proof of principle to validate the data from PCa cell cultures. As shown in Figure 7C, a recruitment profile of BRD4 and H3K27ac comparable with that in PC3 and 22Rv1 cells (Figure 4) was observed in PCa-derived OSC#120 [GS 7 (4 + 3), pT3b] as well as in OSC#97 [GS 6 (3 + 3), pT2c] and OSC#99 [GS 7 (3 + 4), pT2c] (Figure S5), with higher recruitment of BRD4 and H3K27ac in first intron for *CDH1* (site II) and proximal promoter for *ITGB4* (site III) regulatory regions.

## 3. Materials and Methods

### 3.1. Antibody

We used the following materials: β4 integrin (Abcam, Cambridge, UK, #ab133682, RRID:AB_2923284, Western blot dilution 1:1000 and 450-11A, RRID:AB_396065, Western blot dilution 1:500, as in [29,30]), β-actin (Abcam Cat# ab8227, RRID:AB_2305186, Western blot dilution 1:10000), E-cadherin (GeneTex Inc. Alton Pkwy Irvine, CA, USA, Cat# GTX100443, RRID:AB_10729586, Western blot dilution 1:1000 and Abcam, #ab231303, RRID:AB_2923285, Western blot dilution 1:1000), Goat anti-Mouse IgG HRP (Bio-Rad Inc. Hercules, CA, USA, Cat# 170-6516, RRID:AB_11125547, Western blot dilution 1:6000), Goat-anti-Rabbit IgG HRP (SeraCare KPL, Milford, MA, USA, Cat# 5220-0336, RRID:AB_2857917, Western blot dilution 1:8000), IgG (Bethyl Montgomery, TX, USA, Cat# P120-101, RRID:AB_479829, ChIP and RNA-ChIP assays 4 μg/sample), BRD2 (Bethyl Cat#A700–008, RRID:AB_2891809 ChIP assays 4 μg/sample and Active motif Cat#61797, RRID:AB_2793770, ChIP assays 6 μL/sample), BRD3 (Bethyl Cat #A302-368A, RRID:AB_1907251, ChIP assays 4 μg/sample), BRD4 (Cell Signaling Danvers, MA, USA, Cat#13440, RRID:AB_2687578, ChIP and RNA-ChIP assays 1:50, RNA-ChIP assays), H4K12ac (Active motif Carlsbad, CA, US, Cat#39166, RRID:AB_2615076, ChIP assays 6 μL/sample), and H3K27ac (Abcam cat#ab4729, RRID:AB_2118291, Western blot dilution 1:1000, ChIP assays 4 μg/sample).

### 3.2. Cell Cultures and Treatment

The PC-3, Du145, and 22Rv1 cell lines represent models of advanced, androgen-independent prostate cancer with no androgen receptor (AR-null) expression (PC-3, and Du145), or an androgen-insensitive AR variant (ARv7) co-expressed with a full-length AR (22Rv1). The PC-3M-luc2 cell line (PC-3-luc), purchased from Caliper Life# 124089 (RRID: CVCL_5J25), was kindly provided by Prof. Carlo Leonetti (Istituto Nazionale dei Tumori Regina Elena, Rome, Italy). The 22Rv1-luc cell line, generated from 22Rv1 RRID: CVCL_1045, was kindly provided by Prof. Michael Henry (The University of Iowa, Iowa City, IA, USA) as previously described in [30]. The Du145 cell line (RRID: CVCL_0105) was acquired from American Type Culture Collection (ATCC #HTB-81). PC-3-luc was cultured in MEM (Corning, New York, NY, USA, #15-010-CVR), and 22Rv1-luc in RPMI medium (1640 Corning, #10-040-CV), both supplemented with 10% FBS (GIBCO, #10270106) as described in [30]. Du145 was cultured in MEM with 10% FBS (GIBCO, #10270106). The genetic identity of PC-3-luc and 22Rv1-luc cell lines was authenticated by BMR Genomics (Padova, Italy) in October, 2022, as was the Du145 cell line in February, 2023. Mycoplasma contamination was routinely assessed by Indirect (Hoechst) methods and by PCR-based mycoplasma detection (Biontex Laboratories, GmbH, Munich, Germany, UE, # M030-050). PC-3-luc, 22Rv1-luc, and Du145 cells were treated with the following BET inhibitors and degraders, purchased from MedChemExpress: (S)-(+)-JQ-1 (JQ1, Cat. No.: HY-13030), (R)−(−)−JQ1 (R-JQ1, Cat. No.: HY-13030A), dBET6 (Cat. No.: HY-112588), Bi7273 (Cat. No.: HY-100351), OTX-015 (Cat. No.: HY-15743), and LT052 (Cat. No.: HY-130622). Both (S)-(+)-JQ-1 [14] and OTX-015 (Birabresib) [46] are potent and reversible BET (BRD2/3/4) inhibitors with no selectivity between the first (BD1) and second (BD2) bromodomain of the BRD proteins. The (R)-(-)-JQ1 enantiomer is the so-called distomer of (S)-(+)-JQ-1; it is completely inactive, and for this reason it is frequently employed as a negative control of (S)-(+)-JQ-1. LT052 [36] is a highly selective inhibitor of the first bromodomain BD1 of the BET protein BRD4. Bi7273 [35] is a selective and cell-permeable inhibitor of BRD9 and, to a slightly lesser extent, of BRD7. dBET6 [47] is a highly potent, selective, and cell-permeable chemical degrader of BET proteins that exploits the structural moiety of (S)-(+)-JQ-1 as a target-engaging warhead. Compound concentrations causing the inhibition of 50% cell viability (IC50) were determined from the dose–response curves. The cells were then treated using the IC50 dose as follows: JQ1 (0.5 μM JQ1 for PC-3-luc and Du145 and 0.75 μM for 22Rv1-luc), dBET6 (0.125 μM for PC-3-luc and Du145 and 0.1 μM for 22Rv1-luc), OTX (0.5 μM), Bi7273 (5 μM), and LT052 (0.5 μM). R-JQ1 and DMSO were used as negative controls.

Stable *H19* silencing in PC-3-luc and 22Rv1-luc cells was achieved using recombinant GFP-expressing lentiviral vectors [lentiviral vectors for *H19* silencing (siH19, Origene#TL318197V) and scramble vector (Vector, Origene#TR30021)] as previously described in [30].

### 3.3. RNA Extraction, cDNA Preparation, and Real-Time PCR

According to the manufacturer’s instructions, RNA from cells and tissues was extracted using Trizol (ThermoFisher, Waltham, MA, USA). cDNA preparation was performed with a high-capacity kit according to instructions (Applied Biosystems, Foster City, CA, USA), and quantitative real-time PCR using SYBR Green with an evaluation of the dissociation curve were performed as in [30] on the QuantStudio 5 or QuantStudio 7 Pro Real-Time PCR System (Applied Biosystems). The relative amount of each gene was measured as 2^−ΔΔCt^ versus DMSO after normalization with endogenous control (*β-Actin*, *GAPDH*, or *P0*) as in [48]. Primers to *H19*, *CDH1*, *ITGB4*, *P0*, *GAPDH*, and *β-actin* were as in [30], and *CCND1* was as in [29]. The primers used were as follows:

hVIM 5′-CCAAGTTTGCTGACCTCTCTGA-3′ and 5′-GGGACTCATTGGTTCCTTTAAGG-3′; and

hMYC 5′-CTCTGAGGAGGAACAAGAAGATGAG-3′ and 5′-CCAGGAGCCTGCCTCTTTT-3′.

### 3.4. Protein Extraction and Western Blotting

Proteins were extracted and prepared using Trizol (ThermoFisher) with an optimized lysis buffer for the solubilization of Trizol-Precipitated protein as described in [30]. Western blots, performed using 15–20 μg of protein extract, were resolved by 4–12% gradient Invitrogen Precast gel (MES buffer). For H3K27ac detection, proteins were solved by 15% SDS-PAGE. The protein signal was revealed with the ECL Western Blot Detection Kit (Amersham Pharmacia Biotech, Buckinghamshire, UK) using UVIDOC (Eppendorf S.r.l., Hamburg, Germany). Densitometry analysis was performed with ImageJ software (version 1.8.0), [49].

### 3.5. Cell Proliferation Assay

Proliferation was assessed using the IncuCyte system S3 Kinetic Live Cell Imaging System (Sartorius, Essen BioScience, Ann Arbor, MI, USA) according to the manufacturer’s instructions, as described in [30]. Briefly, the cells were seeded in quadruplicate on a 96-well plate (Corning#3688), with IncuCyte readings taken every 2 h and 4 h starting on day 0 (9 or 16 images per well). The images were analyzed using IncuCyte Cell-by-Cell software (version 2022b rev2, [50]).

### 3.6. Chromatin Immunoprecipitation (ChIP)

ChIP assays in cell lines and PCa-derived OSCs were performed using specific antibodies to BRD2, BRD3, BRD4, H4K12ac, and H3K27ac as previously described [30,51]. IgG was used as a negative control. Briefly, chromatin solution was precleared by the addition of Protein G (pierce Chemical co, Rockford, IL, USA) for 1 h at +4 °C and incubated with a specific antibody overnight at +4 °C with mild shaking. DNA IP fragments were analyzed in duplicate by qPCR on the QuantStudio 5 and QuantStudio 7 Pro Real-Time PCR System (Applied Biosystems) using the SYBR Master mix (Applied Biosystems, Foster City, CA, USA) with an evaluation of dissociation curves. Standard curves were generated by serially diluting the input (5-log dilutions in triplicate). The specific sequences isolated by immune complexes were normalized to the corresponding DNA input control, and data represented as relative enrichment. Primers for *c-Myc* enhancer were as in [40], and for *IDO1* promoter as in [41]. Primers for *CDH1* and *ITGB4* promoter were as follows:

CDH1prom-(I) 5′-CCGTGCAGGTCCCATAACC-3′ and 5′-CATAGACGCGGTGACCCTCTA-3′ (as in [29]);

CDH1intr1-(II) 5′-TGCATTCCCGGTCTAAGGAA-3′ and 5′-TTCAGTCTCCTTTCTCATTTTATTGG-3′;

CDH1intr2-(III) 5′-TGGGCAAGCTCCCTCCTT-3′ and 5′-GATCCCCAAATCTGCGTAAATT-3′;

ITGB4prom1-(I) 5′-CCGTAGTTCTCGTTCATCTTGGT-3′ and 5′-TCCTCATGTGGCCTCCAGTAG-3′;

ITGB4prom2-(II) 5′-CTGGCCTGACACACACAGATCT-3′ and 5′-TTTGGGAACAATGTGGAAGGA (as in [30]);

ITGB4prom3-(III) 5′-TGACCTGAACACCCGTGGTA-3′ and 5′-GCACTCGATGCCTTGTTACAGT-3 (as in [29]);

ITGB4intr1-(IV) 5′-CCCATCATGGCGCATCTAAT-3′ and 5′-GGAGCCAATGTTAGAAAGAACGA-3′.

### 3.7. RNA-Chromatin Immunoprecipitation (RNA-ChIP)

RNA-ChIPs were performed with an RNA-ChIP kit (Active Motif) using specific antibodies to BRD2, BRD3, BRD4, and IgG as negative controls as in [29], with some modifications. Briefly, the specific RNA sequences isolated by the immune complexes were subjected to retro-transcription and cDNA preparation using the high-capacity kit (Applied Biosystems) according to instructions. The PreAmp step was performed using 5 μL of cDNA, SYBR green reaction (Applied Biosystems), and specific primers at 40 nM for 14 cycles at 95 °C for 15 s and 58 °C for 4 min. Two microliters of preAmp (1:10 dilution) was used to perform real-time PCR using the SYBR Green Master mix with the evaluation of dissociation curves. Primers for *H19* and *P0* were as in [30], for *MALAT1* as in [44], for *NEAT1v2* and *CHMP2A* as in [42], and for *DEANR1* as in [43].

### 3.8. Subcutaneous Murine Xenograft Model

NOD/SCID (RRID: IMSR_JAX:001303) mice from Charles River Laboratories were housed in 3–4 for cages in a room at a controlled temperature, constant humidity, and a 12 h light/dark cycle with free access to food and water. The standard xenograft mouse model was generated as described in [30]. Briefly, 5/6-week-old male NOD-SCID mice were subcutaneously injected with 3 × 10^6^ cells/mouse with matrigel (1:1). The mice were randomly divided into two groups and treated with JQ1- (25 mg/Kg via IP 5 days/week) or vehicle-treated (DMSO) starting from day 0. Tumor growth was monitored by bioluminescence imaging (IVIS II Lumina, PerkinElmer Italy S.p.A., Milan, Italy) and by digital calipers [tumor volumes: V = (w^2^ × l)/2; w = width, l = length]. At the end of the experiments, subcutaneous tumors were collected for Western blot analysis.

### 3.9. Organotypic Slice Cultures (OSCs)

PCa patients (n = 25) enrolled at the Urology Department of Università Cattolica (Rome, Italy), with the following inclusion criteria, underwent prostatectomy, and fresh explants of tissues were used to generate OSCs as previously described in [29,30,44,52]: (i) clinically localized PCa at diagnosis and (ii) an absence of hormone treatment/radiotherapy before surgery. Notably, all OSCs were evaluated by the pathologist on the original histopathological slide for morphology, tissue architecture, and mass of tumor (≥75%). Briefly, fresh tissues were cut into thick slices (350 μm) using McILWAIN TISSUE CHOPPER (Campden Instruments, Loughborough, England) and cultured at a liquid–air interface using semi-porous tissue culture inserts (PICM03050, Millipore Darmstadt, Germany) placed in a six-well culture plate, using 5 slices/inserts. In total, 22 OSCs (detailed in Table 1) were treated with JQ1 (3 µM) or dBET6 (2 µM) for 48–72 h, and RNA was extracted with Trizol and analyzed as previously described [30]. Three additional OSCs were crosslinked and used for ChIP assays as in [51].

### 3.10. Statistical Analysis

The data were expressed as the mean ± SEM or as the fold change (mean ± SEM), as indicated in the figure legend. The differences among ≥3 groups were analyzed with a Kruskal–Wallis test, and post hoc comparison was performed using the Mann–Whitney U test (α = 0.05). The differences among 2 groups were analyzed with the Mann–Whitney U test using GraphPad Prism 8.0.2 statistical software (GraphPad Prism version 8.0.2 for Windows, GraphPad Software, Boston, MA, USA, www.graphpad.com [53]). The chi-square test was used to compare the proportion of the OSC with at least a 25% reduction in *CDH1* or *ITGB4* after JQ1 treatment between groups, as indicated in the figure legend. *p*-values of <0.05 were considered significant.

## 4. Discussion

Our study provides novel insight into the epigenetic regulation of metastatic dissemination in prostate cancer (PCa), identifying the BET family protein BRD4 as a key driver of the *H19*/cell adhesion molecule axis that promotes collective cell migration. Notably, this regulatory mechanism is active regardless of androgen receptor (AR) status, expanding its therapeutic relevance to castration-resistant (CRPC) and AR-null PCa subtypes, for which treatment options remain limited [17,20,21,22,23].

Mechanistically, we demonstrate that the silencing of the long non-coding RNA *H19* enhances BRD4 recruitment at the promoters and regulatory regions of E-cadherin (*CDH1*) and β4 integrin (*ITGB4*)—two hallmark adhesion molecules associated with the cohesive metastatic phenotype [6,7,8,28,29,30]. This recruitment occurs predominantly at chromatin regions enriched in H3K27 acetylation, consistent with BRD4’s role as a reader of acetylated histones and a facilitator of transcriptional activation [9,10,38,39].

Importantly, *H19* and BRD4 were found to directly interact on chromatin, as revealed by RNA-ChIP assays, highlighting a novel molecular mechanism in which *H19* acts as a negative modulator of BRD4 genomic occupancy. Under physiological conditions, *H19* likely acts as a scaffold or decoy that limits BRD4 recruitment to adhesion gene loci. Its downregulation—such as under estrogen or hypoxic signaling [28,29]—removes this restraint, allowing BRD4 to activate a transcriptional program that favors collective cell migration [6,30].

This is the first study demonstrating that BRD4 promotes the collective migration phenotype in prostate cancer through a mechanism dependent on *H19*. Previous evidence had identified BET family proteins as regulators of integrin signaling in triple-negative breast cancer and non-small cell lung cancer [54,55], but the contribution of non-coding RNAs such as *H19* to this regulatory axis was not explored. Our work now places *H19* at the center of this epigenetic switch, linking environmental cues to chromatin remodeling and transcriptional reprogramming.

Interestingly, while BRD4 has been classically associated with the promotion of epithelial-to-mesenchymal transition (EMT) and tumor invasion [56,57,58], our findings reveal a distinct role for BRD4 in maintaining E-cadherin expression and supporting epithelial features in PCa. This context-specific behavior aligns with previous reports describing the dual function of BRD4 as both an oncogenic driver and a context-dependent transcriptional modulator [59,60,61]. Rather than promoting EMT, BRD4 appears to enforce a collective migration phenotype in the prostate tumor setting—a cohesive strategy associated with high metastatic potential [7,8].

Therapeutically, our results highlight the *H19*–BRD4–adhesion gene axis as a targetable vulnerability in PCa. The pharmacological inhibition of BET proteins using pan-BET inhibitors (JQ1, OTX015) or degraders (dBET6) effectively reduced *CDH1* and *ITGB4* expression and suppressed proliferation across multiple cell models, including AR-positive (22Rv1) and AR-null (PC-3, Du145) lines [12,13,17,20]. Notably, these effects were also observed in vivo in xenograft models and patient-derived organotypic slice cultures (OSCs), where JQ1 treatment significantly downregulated adhesion molecule expression in tissue from patients who later experienced recurrence after surgery.

However, the use of BETis does not have caveats. While initial enthusiasm around JQ1 was driven by its pan-BRD2/3/4 inhibition, recent reports suggest that JQ1 can also exert BET-independent effects, such as promoting metastasis through FOXA1 activation [62]. This effect underscores the importance of refining BET-targeting strategies. In this regard, our study provides encouraging data on the efficacy of other BETis (e.g., dBET6) or the bromodomain 1 (BD1)-selective LT052 capable of disrupting the *H19*/adhesion program and controlling proliferation. These data suggest that domain-selective BETi might offer enhanced specificity with reduced off-target risks [36,47].

Additionally, ChIP analyses performed in both cell lines and OSCs confirmed strong BRD4 recruitment at regulatory regions of *CDH1* and *ITGB4*, further supporting the clinical relevance of this axis. These data suggest that *H19*-mediated control of BRD4 is not a cell-line-restricted phenomenon, but is preserved in human tissues, reinforcing the translational significance of our findings [29,30,34,44,45].

Perhaps most compelling is our observation that OSCs from patients with postoperative recurrence showed greater sensitivity to the JQ1-induced downregulation of *CDH1* and *ITGB4* than those without recurrence. This observation opens up the possibility of using ex vivo BETi response profiles as a biomarker to stratify recurrence risk and guide post-surgical treatment decisions [34].

Taken together, our findings expand the biological role of BRD4 in prostate cancer metastasis and introduce a novel regulatory axis involving *H19* as a critical modulator of adhesion gene transcription. Disrupting this circuit using clinically actionable BET inhibitors offers a promising strategy to intercept collective cell migration, especially in AR-independent settings where current therapies fail.

In conclusion, our study identifies a novel and clinically relevant regulatory axis in metastatic prostate cancer, wherein BET family proteins—particularly BRD4—function as central epigenetic effectors of the *H19*/cell adhesion molecule circuitry. Through direct chromatin binding and interaction with *H19*, BRD4 sustains the transcription of *CDH1* and *ITGB4*, promoting collective cell migration—a metastatic program increasingly recognized in epithelial tumors. Significantly, this BRD4-driven mechanism operates independently of androgen receptor (AR) status, expanding its relevance to AR-null and castration-resistant prostate cancer (CRPC) subtypes currently underserved by standard therapies.

## 5. Study Limitation

Several limitations should be acknowledged. First, the precise molecular mechanism by which *H19* interferes with BRD4 chromatin binding remains incompletely understood. Second, while OSCs offer a patient-derived model that preserves tumor architecture, inter-sample variability may affect the generalizability of findings. Third, BET inhibitors have broad epigenetic activity, and off-target effects cannot be excluded. Finally, although we observed promising results in xenografts and OSCs, in vivo studies evaluating the metastatic potential under BETi treatment are warranted to assess therapeutic efficacy more comprehensively.

## Figures and Tables

**Table 1:** Clinical and pathologic features of PCa patients.

*PCa Patients*	AGE	PSA	Pathologic Gleason Score	Pathologic Stage	Recurrence	Time of Recurrence After Surgery (Months)
*OSC 54*	69	2.4	7 (3 + 4)	pT3a pNx pMx	yes	11
*OSC55*	75	7.1	7 (3 + 4)	pT3a pN0 pMx	yes	4
*OSC 57*	80	15.2	9 (4 + 5)	pT3b pN0 pMx	yes	3
*OSC 58*	76	6,7	7 (3 + 4)	pT3a pN1 pMx	yes	6
*OSC 61*	76	6.1	7 (3 + 4)	pT3b pNx pMx	-	-
*OSC 62*	65	7.6	7 (4 + 3)	pT2c PNx pMx	yes	23
*OSC 63*	70	8	7 (3 + 4)	pT2c pNx pMx	-	
*OSC 64*	65	13	7 (3 + 4)	pT3a pN0 pMx	yes	13
*OSC 67*	69	9	7 (3 + 4)	pT2c pN0 pMx	-	-
*OSC 70*	64	6.7	7 (3 + 4)	pT2c pNx pMx	-	-
*OSC 71*	71	4.8	7 (3 + 4)	pT2c pN0 pMx	-	-
*OSC 74*	69	3.9	7 (3 + 4)	pT2c pNx pMx	-	-
*OSC 75*	78	17	7 (4 + 3)	pT3a pNx pMx	yes	8
*OSC 76*	74	7	7 (4 + 3)	pT2c pN0 pMx	-	-
*OSC 88*	72	9.8	7 (3 + 4)	pT2c pNx pMx	-	-
*OSC 94*	68	3.6	7 (3 + 4)	pT2c pNx pMx	yes	5
*OSC 95*	61	5.4	7 (4 + 3)	pT2c pN0 pMx	-	-
*OSC 98*	65	11	7 (3 + 4)	pT3a pNx pMx	-	-
*OSC 105*	63	21	7 (3 + 4)	pT2c pN0 pMx	-	-
*OSC 110*	65	10	7 (4 + 3)	pT3b pNx pMx	-	-
*OSC 112*	75	23	9 (4 + 5)	pT3a pNx pMx	-	-
*OSC 114*	60	11	7 (4 + 3)	pT3a pN1,pMx	yes	3

## Data Availability

The original contributions presented in this study are included in the article/Supplementary Materials, and further inquiries can be directed to the corresponding authors.

## Supplementary Materials

The following supporting information can be downloaded at: https://www.mdpi.com/article/10.3390/ncrna11030033/s1, Figure S1: Effect of several BET family inhibitors, BRD2/3/4 or BRD7/9 members, on proliferation in 22Rv1 cell line. Figure S2: Effect of LT052 on *CDH1* and *ITGB4* mRNA level and cell proliferation. Figure S3: Effect of JQ1 treatment on H3K27ac level in vitro and in subcutaneous murine xenograft tumor. Figure S4: BRD2, BRD3, BRD4, and H3K27ac recruitment onto *c-Myc* enhancer and IDO1 promoter. Figure S5: In vivo ChIP assays on organotypic Slice cultures.
