# Supplementary material for: Bromodomain and Extra-Terminal Family Proteins BRD2, BRD3, and BRD4 Contribute to H19-Dependent Transcriptional Regulation of Cell Adhesion Molecules, Modulating Metastatic Dissemination Program in Prostate Cancer
Source: Noncoding RNA. 2025 Apr 29;11(3):33. doi: 10.3390/ncrna11030033 (PMC12101203; doi:10.3390/ncrna11030033)
Supplement: Supplementary file 1 [file ncrna-11-00033-s001.zip › ncrna-3489913-supplementary.pdf]

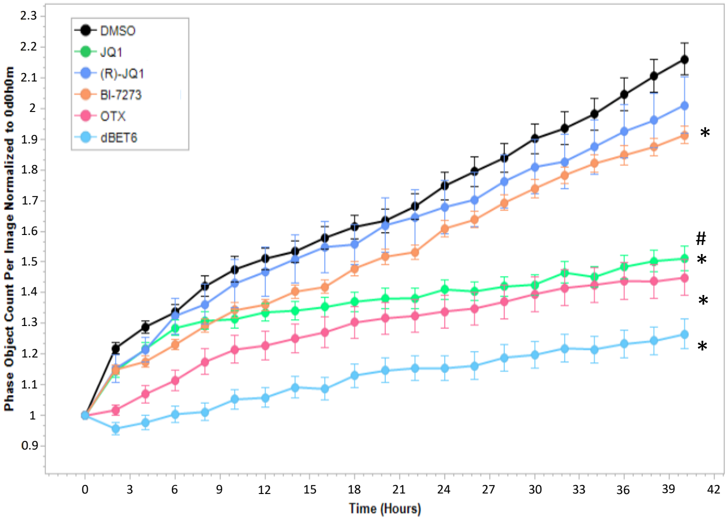

Figure S1. Effect of several BET family inhibitors, BRD2/3/4 or BRD7/9 members, on proliferation in 22Rv1 cell line. *Cells were treated with JQ1, Bi-7273, OTX or dBET6 and DMSO or (R)-JQ1 as a control (as in described in legend to Figure 3). Cell proliferation was monitored using the IncuCyte live cell analysis system. Cell confluence was calculated from raw data images; the data shown is a representative experiment of 4 biological replicates, and each time point represents the mean of 4 samples. *P < 0.05 vs. DMSO; # <0.05 vs. (R)-JQ1.*

A

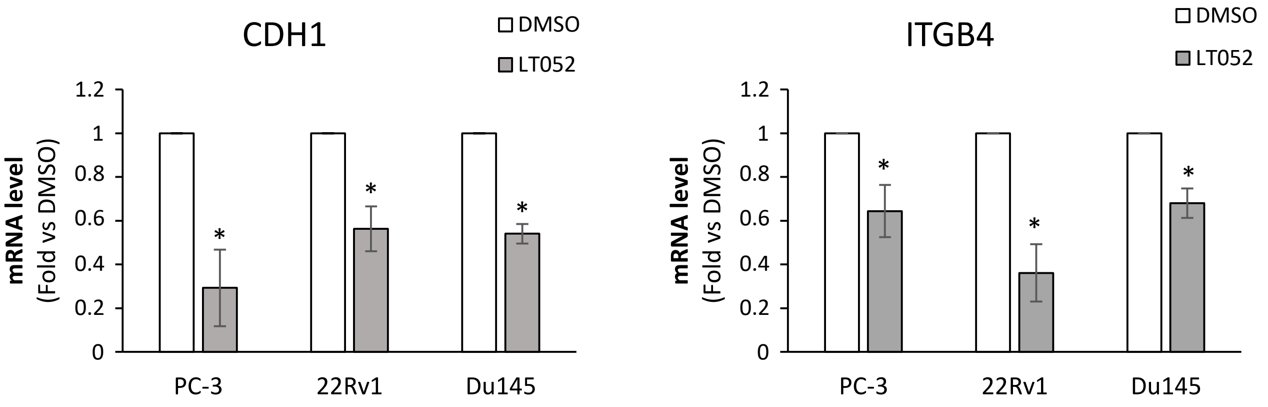

B

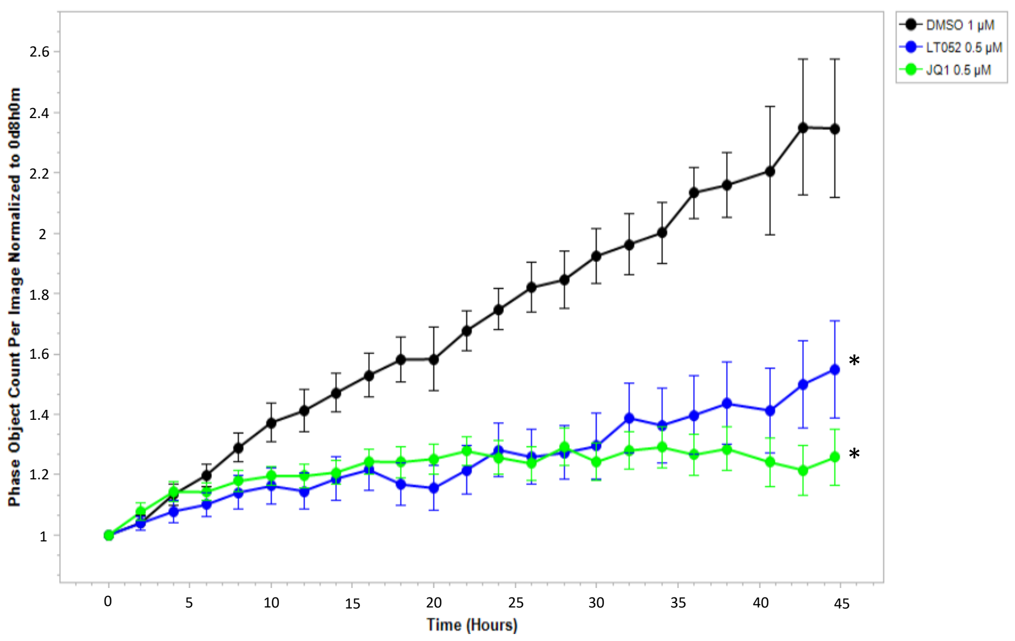

Figure S2. Effect of LT052 on CDH1 and ITGB4 mRNA level and cell proliferation. *A) E-cadherin (CDH1), β4 integrin (ITGB4) mRNAs were assessed by qPCR in PC-3-luc (PC-3), 22Rv1-luc (22Rv1) and Du145 after 72h treatment with LT052 or DMSO as control. Data, plotted as fold change vs. DMSO, represent the mean ± SEM of 3-4 independent experiments. * P <0.05 vs. DMSO. B) PC-3 cell proliferation was monitored using the IncuCyte live cell analysis system. Cell confluence was calculated from raw data images; the data shown is a representative experiment of 4 biological replicates, each time point represent the mean of 4 samples.*

A

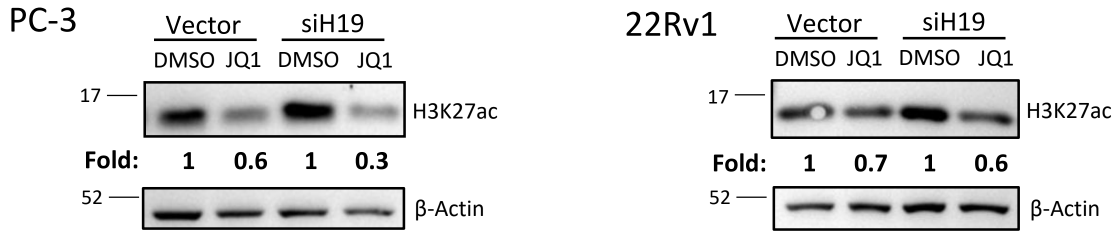

B

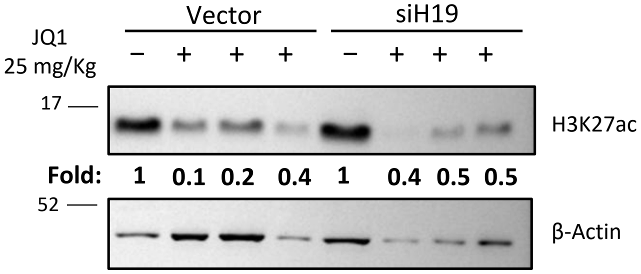

Figure S3. Effect of JQ1 treatment on H3K27ac level in vitro and in subcutaneous murine xenograft tumor. *A) Representative H3K27ac western blot in in PC-3-luc (PC-3, left) and 22Rv1-luc (22Rv1, right) after stable H19 silencing (siH19) compared to control vector (Vector) in presence or absence of JQ1 or DMSO as control. B) H3K27ac level analyzed by western blot in tumor samples of xenograft implanted PC-3-luc siH19 or Vector cell as described in the legend to Figure 2. β-Actin was used as a loading control. Molecular weight marker is indicated. Number represents densitometric analysis of protein level normalized to β-Actin and expressed as fold change vs. DMSO.*

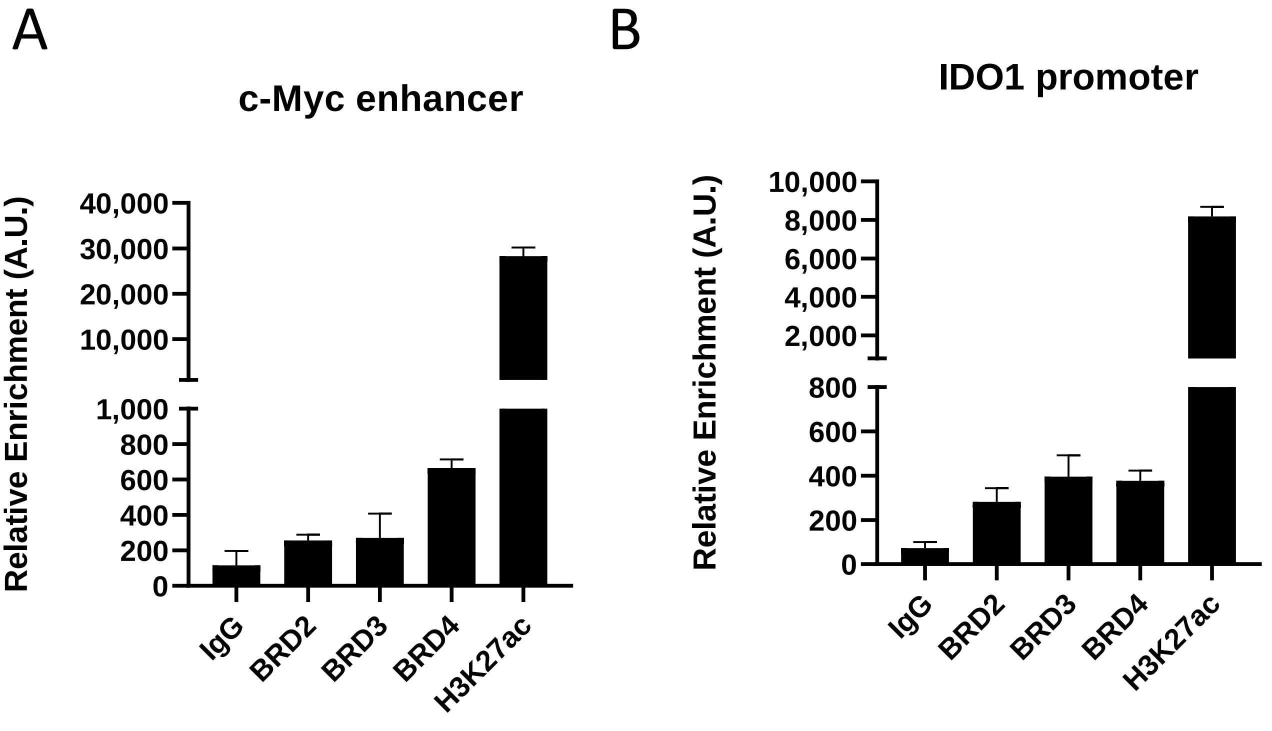

Figure S4. BRD2, BRD3, BRD4, and H3K27ac recruitment onto c-Myc enhancer and IDO1 promoter. *Recruitment of BRD2, BRD3, BRD4, and H3K27ac on the regulatory region of c-Myc (A) and IDO1 (B) genes by ChIPs in PC-3 cells. IgG served as a negative control. Values represent mean ± SEM of three independent experiments.*

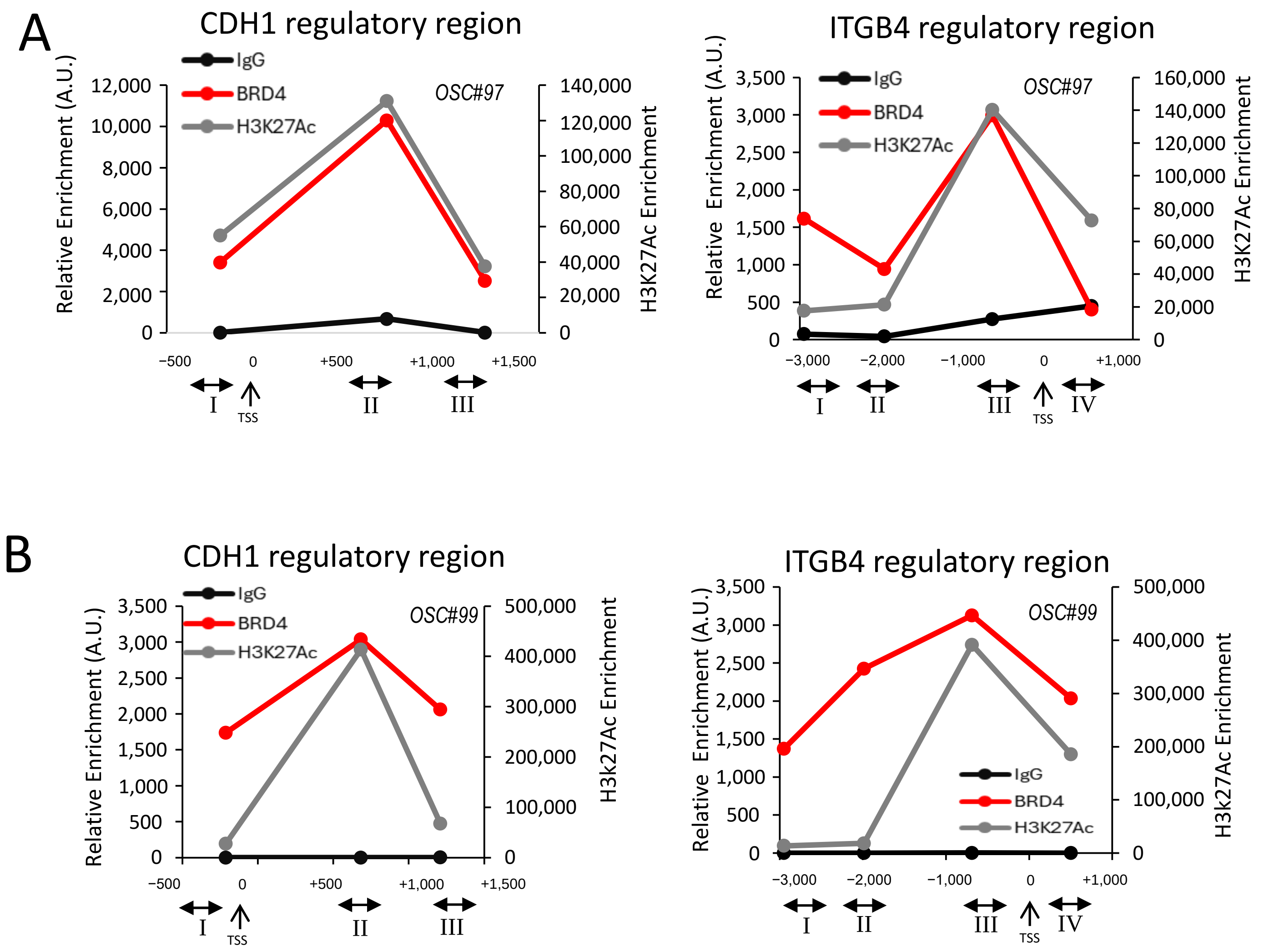

Figure S5. In vivo ChIP assays on Organotypic Slices Cultures. *A, B) In vivo ChIP assays were performed using fresh OSCs, OSC#97 (A) and OSC#99 (B), as in the legend in Figure 7. Briefly, immunoprecipitations were with antibodies to BRD4 and H3K27Ac or IgG as negative control. Recruitment onto the CDH1 and ITGB4 regulatory regions was detected by quantitative PCR (qPCR) using primers for regions I, II, III, and IV, as in the legend in Figure 4.*
